# Teaching the physiology of the human body in non-formal spaces: pilot experience of a Service-Learning methodology and the interaction between students of different educational levels

**DOI:** 10.3389/fphys.2023.1268766

**Published:** 2023-10-10

**Authors:** Laura García-Durán, Silvia Claros, Pablo Zamorano-González, Marta González-García, Laura Carrillo-Franco, Marina Ponce-Velasco, Belén Gago, María García-Fernández, Manuel Víctor López-González, Ana Aiastui

**Affiliations:** ^1^ Department of Developmental and Educational Psychology, Universidad de Málaga-IBIMA Plataforma BIONAND, Málaga, Spain; ^2^ Department of Human Physiology, Universidad de Málaga-IBIMA Plataforma BIONAND, Málaga, Spain; ^3^ Department of Cell Biology, Universidad de Málaga-IBIMA Plataforma BIONAND, Málaga, Spain; ^4^ Multidisciplinary 3D Printing Platform, Biodonostia Health Research Institute, San Sebastián, Spain

**Keywords:** Service-Learning, outreach, non-formal education, undergraduate students, secondary education students, workshop, community service

## Abstract

Research institutes and universities have strengthened the development of biomedicine outreach activities, constituing a non-formal education system of science literacy, although with little commitment from undergraduate students. However, as a Service-Learning methodology, these outreach activities could work as a tool for the acquisition of skills by students of Health Science Degrees. Described here is the development of the workshop entitled “Exploring the human body” at the Biodonostia Health Research Institute and the pilot experience of its implementation as a Service-Learning activity at the University of Málaga. Firstly, 359 secondary education students were mentored by Ph.D. students through a 5-station workshop with experiments and activities related to the physiology of the human body. Then, 301 undergraduate students of Medicine and Nursing Degrees advised 965 secondary education students. Both groups of students assessed the workshop via questionnaires and a debriefing. The data showed an overall score of 4.6 out of 5 for the workshop. Undergraduate students reported a positive impact on their academic background (4.8 out of 5), mainly due to the improvement of oral communication skills (34%). Therefore, this methodology could be a valid and applicable tool to develop the cross-disciplinary competences of undergraduate students.

## 1 Introduction

The scientific and technological literacy of students, and their knowledge acquisition of scientific terms, laws and theories useful in their daily and work life, and for the consolidation of citizenship, has long been an important goal for teachers (Acevedo et al., 2003).

This knowledge could be obtained through formal or non-formal educational systems. Outreach activities are included in non-formal education. The aim of these initiatives is to communicate scientific advances in a specific field to the general population, using adapted language to set out the ideas at a level suited to the knowledge, interest and needs of the non-scientific audience (Olmedo Estrada, 2011). However, they could also be used as a complementary tool to formal education in schools and high schools, to inspire and encourage professional vocations and develop a scientific culture in children and teenagers (Olmedo Estrada, 2011). Moreover, these activities could be used to address and reduce the STEM gender gap (Levine et al., 2015), which seems to be highly influenced by secondary schools context (Legewie and Diprete, 2014).

In recent decades, the outreach activity programs developed by research institutes and universities have proved essential to build bridges between scientists and society, which include the relationship between lecturers and scientists and both primary and secondary school teachers and students. This type of activity, when organized by universities involve mostly graduate students and to a lesser extent undergraduate students, under the supervision of professors (Demb and Wade, 2012; Curtis, 2017). However, studies suggest that the involvement of undergraduate students in STEM outreach activities allows them to apply their knowledge and acquire skills and cross-disciplinary competences such as oral communication and self-esteem (Brownell et al., 2013; Kompella et al., 2020; Murphy and Kelp, 2023).

Furthermore, the use of the Service-Learning methodology could also contribute to the acquirement of such skills by students of Health Science Degrees. This educational methodology combines academic learning of content, skills and competences with reflection and real-world experiences of service to the Community (Seifer, 1998). Due to the potential benefits of this “learning by doing” or “learning through teaching” methodology, the American Psychological Association (APA) recommended, in 2010, the engagement of undergraduate students in experiences that contribute to applying what they learn, such as volunteer activities and Service-Learning (Halpern, 2010). In fact, several studies describe how significant learning experiences have been successfully developed undertaken with Medicine and Nursing students in the field of physiology, involving a very specific issue such as kidney disease (Reddix, 2023), pharmacology (Husaini et al., 2022), the physiology of drug addiction (Lichtenberg et al., 2020) and the promotion of healthy habits (Glòria et al., 2022). These activities, consisted in the development of lessons and demonstrations, aiming to enhance the interest and understanding of the students on health issues and increase their knowledge and their communication skills, among others.

However, to our knowledge, no activities have been develop to increase the interest in the study and research of the physiology taking into account different biological levels and that would include hands-on activities for secondary school students. Therefore, to find ways to increase the involvement of undergraduate students, mainly females, in biomedicine outreach activities with a social perspective, we designed a workshop entitle “Exploring the human body.” It is a novel broad program, which includes several hands-on activities, focusing on the physiology of biomolecules, cells, tissues, organs, and systems levels.

This paper sets out its development at the Biodonostia Health Research Institute (San Sebastián, Northern Spain) and therefore, the pilot experience of implementing it as a Service-Learning activity was analyzed, in which undergraduate students of Medicine and Nursing Degrees at the University of Málaga (Málaga, Southern Spain), enrolled in the courses Physiology and Pathophysiology, participate as mentors to secondary education students. The final aim of this activity would be to promote science culture in biomedicine among young people, detect differences between genders, and to palliate the shortage of economic and material resources in high schools, as well as to analyze if it could be useful for the students to develop cross-disciplinary skills.

## 2 Material and methods

### 2.1 Participants

Secondary education students (15 or 17 years old) from high schools in the provinces of Guipúzcoa (northern Spain; school years: 2014–2015 to 2015–2016; *n* = 359) and Málaga (southern Spain; school years: 2016–2017 to 2019–2020 and 2021–2022 to 2022–2023; *n* = 965) participated in the workshop. Their sciences teachers accompanied them to the facilities of the institutions.

Undergraduate students of Nursing Degrees at the University of Málaga (*n* = 301; female *n* = 248; male *n* = 53) enrolled in Physiology and Pathophysiology courses were engaged in the workshop as mentors. Student participation was voluntary, and they could obtain an extra 5% on their final score.

### 2.2 Workshop and Service-Learning methodology

The workshop lasted 4 h and was carried out at the facilities of either the Biodonostia Health Research Institute (San Sebastián, Spain) or the University of Málaga (Málaga, Spain). The secondary education students were divided into 5 groups (five to seven students per group) and mentored by 2 people throughout the 5 stations, each lasting 30 min. The hands-on activities included at each station were designed to teach and learn how the physiology of biomolecules, cells, tissues, organs, and systems can be studied.

Before the workshop, mentors were taught in a 2-hour preparatory session to use active learning strategies to explain the activities and how to facilitate each one.

During the development of the workshop, two lectures or researchers of the institutions supervised the mentors to solve their questions and doubts so the information that the secondary students in every session is uniform and correct.

### 2.3 Assessment

At the end of the workshop, a questionnaire was submitted to the secondary education students to rate each of the stations (using a numerical scale from 1 to 5, with 5 being the highest score), highlight 3 things they had learnt, and add some suggestions and comments (Supplementary Tables S1, S2). Accompanying science teachers were informally asked to give their opinion about the workshop.

The undergraduate students were also asked to fill out a self-constructed survey created using a Google form, as a way to reflect upon these experiences, rate the activity and each of the workstations, and obtain information about their participation, the academic competences and their personal background (Supplementary Tables S3, S4). This is a self-constructed questionnaire, based on that developed by Solanes Puchol et al., 2012 for generic competencies in undergraduate students. The questionnaire was evaluated by experts in science didactic and assessment systems from the University of Málaga (Cronbach’s alpha yields a value of 0.8377, which indicates a high internal consistency). It included a number of quantitative questions: some were designed to give ‘yes’ or ‘no’ answers, some used a five-point Likert scale, which included the numerical scale from 1 to 5, with 5 being the highest score, and some were open-ended or multiple choice questions.

### 2.4 Data analysis

The questionaries’ responses were collected using a direct administration procedure, which provided a high response rate (Fraenkel and Wallen, 2009). Data (punctuation values) for each five-points Likert scale question was expressed as mean ± standard error of the mean (SEM). The statistical analysis was performed using GraphPad Prism 9.5.1 (GraphPad Software; La Jolla, CA, United States; www.graphpad.com), setting statistically significant differences at *p* < 0.05. Data normality was established by the Kolmogorov-Smirnov test. One-way ANOVA on ranks (Kruskal–Wallis test) followed by *post hoc* Dunn’s test was performed to establish differences between the rating of each station in either total, female students or male students groups. Mann-Whitney *U* test was used to establish significant differences between male and female answers.

### 2.5 Bioethics

The study was reviewed and approved by the Ethics Committee of Research of the University of Málaga (111-2023-H). All the participants provided their written informed consent to participate in this study.

## 3 Results

### 3.1 Development of the outreach workshop

#### 3.1.1 Workshop

The hands-on activities of the workshop were designed by senior researchers at the facilities of the Biodonostia Health Research Institute with expertise in physiology and cell biology fields, under the supervision of Dr. Belén Gago and Dr. Ana Aiastui. Fifteen Ph.D. students and young researcher participated as mentors and were instructed in the procedure for each station.

**FIGURE 1:**
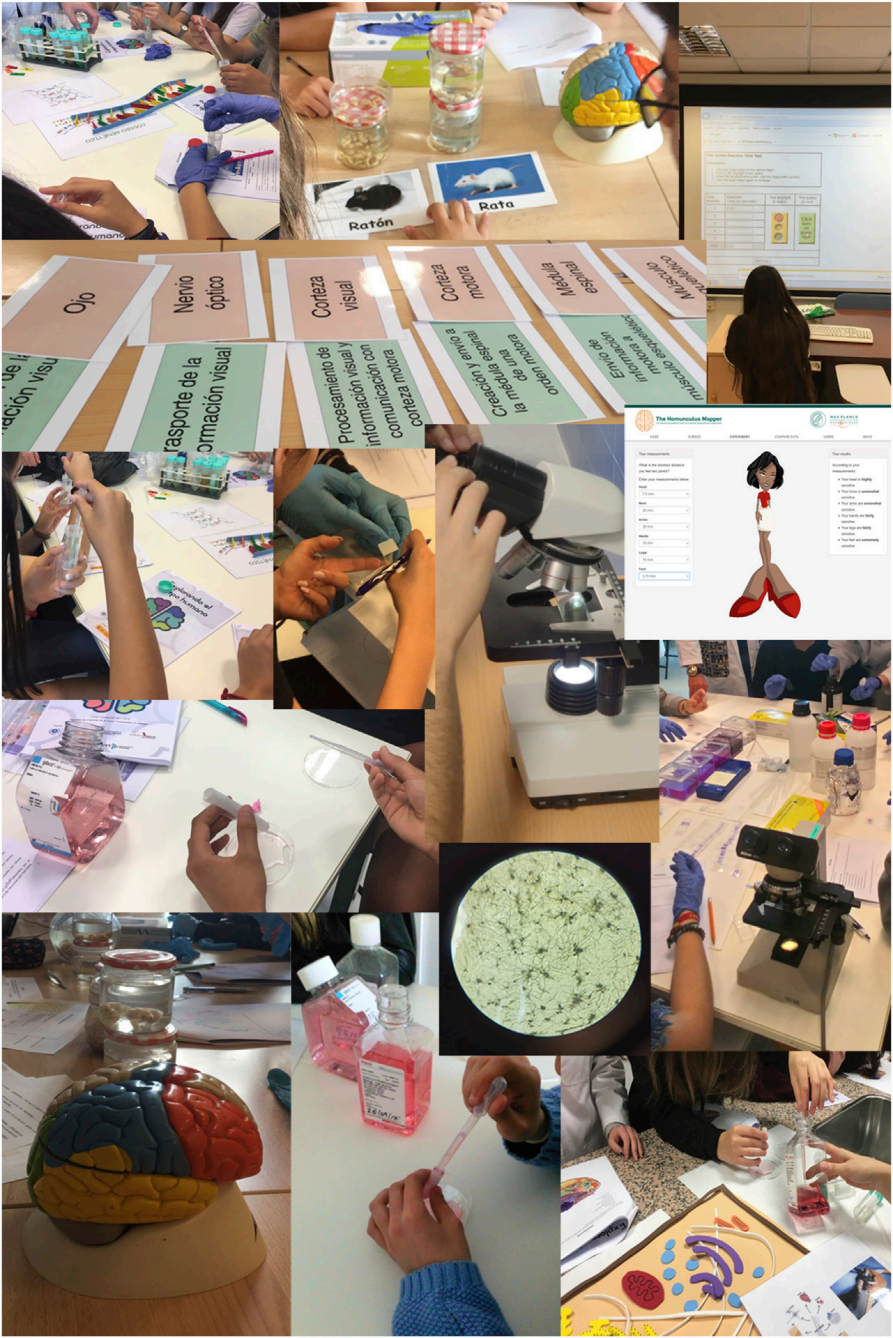
Photographs of the material used in the stations of the workshop “Exploring the human body”.

At the first station, which addressed biomolecules, a DNA extraction of buccal cells was performed and the effects that mutations in this structure may have on health are explained, using several diseases as examples. Students are then asked to design an animal eukaryotic cell using pieces of foam as organelles, at the cells workstation, where stem cells were also explained and students performed a simulation of bone-marrow-derived stem cell extraction. At the tissues station, a histological staining protocol using cresyl violet was carried out on rat brain sections, the results of which would be compared under the microscope with Golgi stained brain sections. Historical background on scientists was also introduced by the mentors to the students. After this, at the organ station, the students had to figure out which animal each brain in the collection corresponds to, explaining any differences and similarities they find between them. Moreover, the students mapped their sensory cortical homunculus using the webpage https://brainmapper.org/. At the last station, a reaction time game was performed to explain how both neural and musculoskeletal systems interact, and a simulation of myelinated and non-myelinated axons was made by students to describe the propagation of action potentials in fibers. Photographs of the material used in the different stations can be found in Figure 1.

#### 3.1.2 Assessments by secondary education students

At the end of the workshop, secondary education students from the province of Guipúzcoa were asked to evaluate it. The analysis of the responses indicated that the overall rating of the workshop was 4.14 ± 0.08 out of 5 points. The scores for each station of the workshop are included in Table 1. The station rated by both male and female students with the lowest significant score compared to all the other stations was the cell station (*p* < 0.001). The biomolecules, organs and systems stations were similarly rated compared to the cell and tissue stations (*p* < 0.001). No significant differences were observed between male and female students in the score of each station (Table 1).

**TABLE 1:** Score values for each station of the workshop by secondary education students from the province of Guipúzcoa.

	Biomolecules	Cells	Tissues	Organs	Systems	H
Total	4.40 ± 0.04^$^	3.67 ± 0.05^&^	4.01 ± 0.05^%^	4.33 ± 0.05^$^	4.43 ± 0.04^$^	177.2 *p* < 0.001
Male (132)	4.39 ± 0.06^$^	3.59 ± 0.06^&^	3.98 ± 0.08^*^	4.24 ± 0.08^@^	4.37 ± 0.7^$^	69.26 *p* < 0.0001
Female (227)	4.41 ± 0.05^$^	3.71 ± 0.07^&^	4.01 ± 0.06^%^	4.37 ± 0.06^$^	4.45 ± 0.05^$^	109.7 *p* < 0.0001
U (male vs. female)	14.45 *p* = 0.53	13.89 *p* = 0.23	14.68 *p* = 0.73	13.65 *p* = 0.12	14.29 *p* = 0.41	

ANOVA, on ranks followed by *post hoc* Dunn’s test and Mann-Whitney *U* test. ^$^ *p* < 0.001 vs. cells, tissues; ^&^ *p* < 0.001 vs. biomolecules, tissues, organs, systems; *p < 0.001 vs. biomolecules, cells, systems; ^%^ *p* < 0.001 vs. biomolecules, cells, organs, systems; ^@^ *p* < 0.001 vs. cells.

### 3.2 Implementation of the workshop as a Service-Learning activity

Since the workshop was positively assessed and after improving some of the activities at the stations, it was tested on a pilot basis as a Service-Learning activity, involving Nursing Degree students at the facilities of the University of Málaga. They mentored students from secondary education schools in the province of Málaga. The assessment provided by the two groups of students was analyzed.

#### 3.2.1 Assessment by secondary education students

Considering the evaluation of the 5 stations of workshop made every year by each participant, the overall rating was 4.6 ± 0.07. The scores for each station of the workshop are included in Table 2. The station rated with the lowest score was the cell station, by both male and female students, and the difference was significant between stations. No significant differences were observed between male and female students in the score of each station (Table 2).

**TABLE 2:** Score values for each station of the workshop by secondary education students from the province of Málaga.

	Biomolecules	Cells	Tissues	Organs	Systems	H
Total	4.69 ± 0.02	4.22 ± 0.03^&^	4.62 ± 0.03	4.63 ± 0.03	4.68 ± 0.03	235.7 *p* < 0.0001
Male (411)	4.63 ± 0.03	4.24 ± 0.04^&^	4.58 ± 0.03	4.61 ± 0.04	4.68 ± 0.03	88.70 *p* < 0.0001
Female (554)	4.72 ± 0.02	4.12 ± 0.04^&^	4.65 ± 0.02	4.65 ± 0.02	4.67 ± 0.02	148.7 *p* < 0.0001
U (male vs. female)	108.64 *p* = 0.10	112.99 *p* = 0.83	108.54 *p* = 0.12	110.37 *p* = 0.29	112.50 *p* = 0.68	

ANOVA, on ranks followed by *post hoc* Dunn’s test and Mann-Whitney *U* test. ^&^ *p* < 0.001 vs. biomolecules, tissues, organs, systems.

Among the open comments made by the students, the following should be highlighted: “It should last longer,” “I would like to participate in it again”, “It should be done more frequently, so we could learn from practice,” “The mentors explained everything perfectly,” “More activities like this should be done at the University,” “The workshop has far exceeded my expectations,” “I am thinking of studying a degree related to the biomedicine field.”

It should be added here that secondary school teachers, who supervised and accompanied the students, emphasized that the activities of the workshop have reinforced the knowledge they explain in class and they highly valued that the workshop takes place at the laboratory facilities of the University.

#### 3.2.2 Assessment by undergraduate students

The overall rating of the stations of the workshop was 4.64 ± 0.1 out of 5 points. The results of the responses evaluating the stations of the workshop are shown in Table 3. The station rated with the lowest score was the station “cells,” by both male and female students, and the difference was significant compared to the other stations. Meanwhile, the highest rated station was the station “biomolecules,” mainly by female students (Table 3). No significant differences were observed between male and female students in the score value of each station (Table 3).

**TABLE 3:** Score values for each station of the workshop by undergraduate students of medicine and nursing degrees.

	Biomolecules	Cells	Tissues	Organs	Systems	H
Total	4.85 ± 0.02^#^	4.23 ± 0.05^&^	4.64 ± 0.04	4.73 ± 0.03	4.60 ± 0.04	146.4****
Male (53)	4.81 ± 0.06	4.25 ± 0.11	4.62 ± 0.09	4.68 ± 0.07	4.55 ± 0.09	20.07***
Female (248)	4.86 ± 0.02^#^	4.22 ± 0.05^&^	4.65 ± 0.04	4.75 ± 0.04	4.62 ± 0.04	127.6****
U (male vs. female)	6.25 *p* = 0.34	6.57 *p* = 0.1	6.43 *p* = 0.76	5.99 *p* = 0.17	6.23 *p* = 0.46	

ANOVA, on ranks followed by *post hoc* Dunn’s test and Mann-Whitney *U* test. #*p* < 0.001 vs. cells, tissues, systems; ^&^ *p* < 0.001 vs. biomolecules, tissues, organs, systems.

All the students have rated the organization of the workshop and the material used in each station very positively (Table 4), with no significant differences between male and female students.

**TABLE 4:** Score values for the structure and organization and the material used in the workshop by undergraduate students of medicine and nursing degrees.

	Structure, organization	U (male vs. female)	Material used	U (male vs. female)
Total	4.66 ± 0.03		4.82 ± 0.03	
Male (53)	4.68 ± 0.07	6.46 *p* = 0.84	4.77 ± 0.06	6.03 *p* = 0.14
Female (248)	4.65 ± 0.03		4.83 ± 0.03	

Mann-Whitney *U* test. Non-significant differences were found between male and female.

**FIGURE 2:**
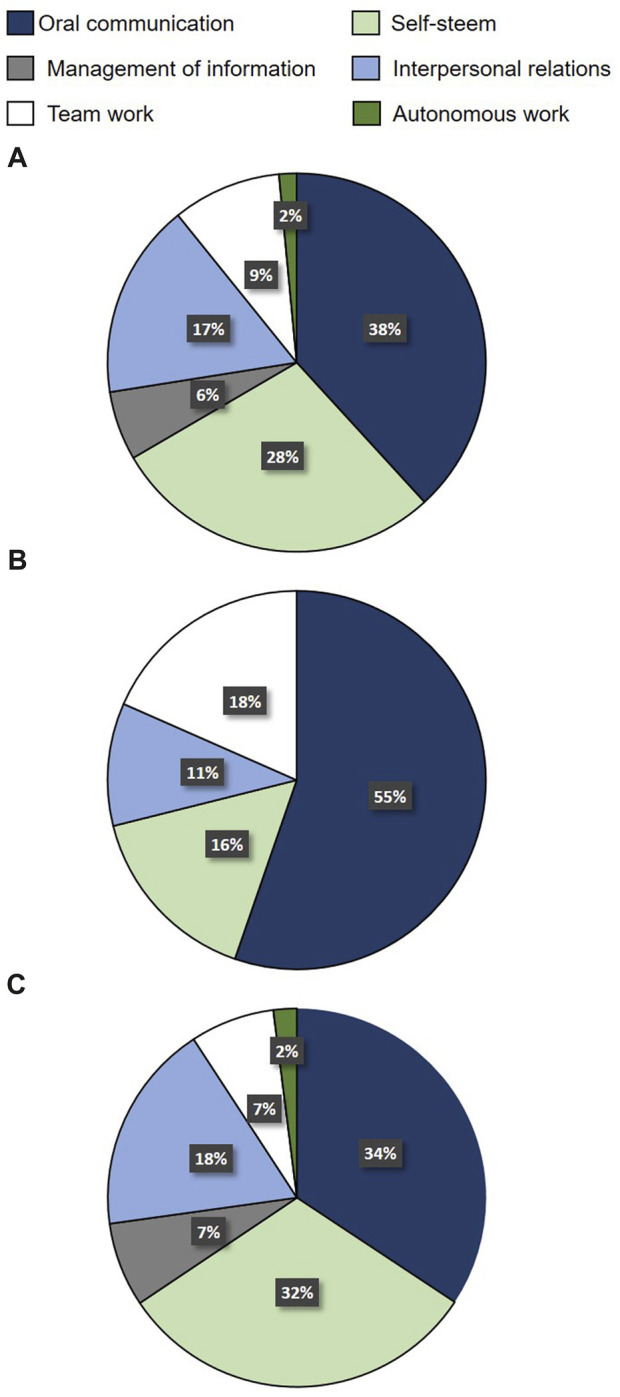
Competences or skill that undergraduate students believe they have improved because of their participation in the workshop, regarding all **(A)**, male **(B)** or female **(C)** students. Values represent percentages of total number of answers for each students group.

To the question about what skill or competence they think they have improved thanks to their participation in the workshop, 38% of the students answered oral communication skills (34% of the female students and 50% of the male students) (Figure 2), followed by self-esteem (28%) and interpersonal relationship (17%).

The students were also asked about their agreement with different statements (Table 5). They manifested a high level of agreement regarding the positive impact that their involvement in the workshop had on their academic background, and the fact that it far exceeded their expectations, with the response of both genders being very similar. They also considered that similar activities should be offered in other courses of the Degree. No differences between male and female students were found regarding these issues.

**TABLE 5:** Score values for questions about the importance of the workshop for undergraduate students of medicine and nursing degrees.

	My enrolment in this activity has a positive impact in my academic background	U (male vs. female)	The workshop has far exceeded my expectations	U (male vs. female)	Similar activities should be develop in our faculty	U (male vs. female)
Total	4.80 ± 0.03		4.78 ± 0.03		4.84 ± 0.03	
Male (53)	4.90 ± 0.04	5.93 *p* = 0.07	4.77 ± 0.06	6.35 *p* = 0.56	4.89 ± 0.06	6.17 *p* = 0.25
Female (248)	4.78 ± 0.03		4.78 ± 0.03		4.83 ± 0.03	

Mann-Whitney *U* test. Non-significant differences were found between male and female.

Regarding the responses to open questions, 100% of the students answered that they had enough knowledge and information to take part in the workshop, meaning that the preparatory session was a useful resource. On the other hand, even though 71.8% of the students have not previously participated in similar activities (72.2% of women and 69.8% of men), 100% of the students indicated that they would like to participate in the workshop again and that they would recommend it to their colleagues. On top of that, 100% of them stated that they would have liked to participate in a similar activity by the time they were in high school.

Among comments about the workshop, the following quotes stand out: “The activities of the workshop were very interesting,” “This workshop should be carried out with younger students, so it could help them choose their academic itinerary in high school,” “Repeat the activity every year!!,” “Increase the number of sessions so more undergraduate students could participate,” “The cell station should be more interactive,” “We should spend more time with secondary education students so they could ask us more questions related to the university admissions process,” “I really enjoyed it!,” “Explain the physiology of other organs,” “The relationship between university and high schools should be strengthened,” “We should do more activities like these in others courses.”

## 4 Discussion

The inclusion of undergraduate students of Health Science degrees in the workshop using the Service-Learning methodology had positive effects on them and their academic background.

The workshop that we was designed is a non-formal educational experience for secondary education students. It had a small group format, which allowed for a low-distraction environment for secondary education students and a high interaction between them and the university students. Furthermore, some secondary education students who may not have considered a college education until now may even be contemplating attending university in the future, as happened in other science dissemination activities (Gilbertson et al., 2022).

The feedback from secondary education students about the workshop was very positive, as they rated the activities carried out at the different stations highly, and commented on what they had learnt, suggesting that it is a successful methodology for non-formal education. A number of previous studies have proven the positive impact that experimentation outreach programs have on primary and secondary education students, improving their attitude towards mathematics (Fernández-Cézar et al., 2020) as well as physiology (Vargas et al., 2016; Hendrickson et al., 2020). In fact, the American Physiological Society (APS) promotes a great variety of outreach activities, especially those focused on secondary school students (Metz et al., 2018; Elmer et al., 2023). Thus, this helps to continue with the workshop, and provides motivation to try to improve them and reach more students, as in fact was done when it began to be carried out at the University of Málaga. The data suggests that there is still room for improvement, as evidenced by the cell station, which received the lowest rating by students even after changing the material used when the workshop was carried out at the University of Málaga facilities. This may be because the activities included in this station are less interactive than those in the others. However, this could be easily corrected in the future.

Another issue to take into account is that there are no differences on the rating of the stations between male and female students from secondary schools. Therefore, the workshop turns out to be equally interesting for both sexes, suggesting that the participation could contribute to increase attitudes towards sciences of female secondary students as in other outreach programs (Barmby et al., 2008).

Service-Learning programs have proved to have significant benefits for students of Health Science degrees, such as practical learning, improved communication skills, the development of empathy or a better understanding of social determinants of health (Marcilla-Toribio et al., 2022; Demirören and Atılgan, 2023). In line with this, the results showed that the undergraduate students state that they improved their communication skills by participating in the workshop. In fact, in the work by Segarra and Tillery, 2018, most of the students expected and looked forward to improving their science communication skills by engaging in an outreach activity. Moreover, the undergraduate students in this study considered that Service-Learning activities are formative, that they enhance their commitment to citizenship and that activities such as the workshop should be done more often, in accordance with previous studies that emphasize the positive effect that being a mentor in outreach programs has on undergraduate students (Diaz et al., 2019; Adams et al., 2020).

Moreover, the Service-Learning methodology is based on the real needs or challenges that can be found in the immediate context of institutions (Seifer, 1998). The large number of secondary education students and high schools that have participated in the workshop is noteworthy, and has increased over the years, addressing their needs and increasing the cooperation between the university and other educational institutions in terms of community partnerships, collaborative working relationships, and social innovation.

Despite the positive results and the number of both secondary education and undergraduate students that have participated, this study has several limitations. Firstly, the participation in the Service-Learning activity is voluntary, due to limitations on the availability of the facilities, and mentors get an extra 5% on their final score. Thus, not all the students enrolled in the courses are able to participate, and those who do are highly motivated *per se*. Secondly, although preparatory session for undergraduate students took place before the workshop, and professors and researchers supervised them to guarantee that information is uniformly given in each session, we cannot rule out mistakes in the explanations. To improve this issue, we should include a test to ensure they correctly gain the knowledge and abilities needed and a second preparatory session for those that would require it. Thirdly, written reflections and semi-structured interviews for the undergraduate students could be included, since they have proven to be a good tool to analyze the influence of the Service-Learning activity on students (Pilling et al., 2021). In addition, the participation of undergraduate students in outreach activities has been shown to increase their interest in physiology and improve their understanding of this scientific field (Borges and Mello-Carpes, 2015; Altermann et al., 2016). Based on that fact, it would be possible to work on a greater involvement of these students in the organization and coordination of the workshop and even work on the idea of new activities that explain other physiological issues using new technologies such as 3D printing. On the other hand, although the involvement in the workshop seems to contribute to the acquisition of communication skills, the manner in which students could develop other competences, such as leadership, could be considered (De Juan Pardo et al., 2022).

In summary, the pilot experience of using an outreach event as a Service-Learning teaching methodology facilitates active learning and skills acquisition and community engagement among university students, since both university and secondary education students reported positive benefits. Furthermore, this educational outreach activity is amenable and extensible to all the branches of science.

## Data Availability

The raw data supporting the conclusion of this article will be made available by the authors, without undue reservation.
