# Supplementary material for: Teaching the physiology of the human body in non-formal spaces: pilot experience of a Service-Learning methodology and the interaction between students of different educational levels
Source: Front Physiol. 2023 Oct 10;14:1268766. doi: 10.3389/fphys.2023.1268766 (PMC10597698; doi:10.3389/fphys.2023.1268766)
Supplement: Supplementary file 1 [file Table1.DOCX]

Supplementary Material

Teaching the physiology of the human body in non-formal spaces: pilot experience of a Service-Learning methodology and the interaction between students of different educational levels

Laura García-Durán^1†^, Silvia Claros^2†^, Pablo Zamorano-González^2^, Marta González-García^2^, Laura Carrillo-Franco^2^, Marina Ponce-Velasco^3^, Belén Gago^2*^, María García-Fernández^2^, Manuel Víctor López-González^2#^, Ana Aiastui^4*#^

*** Correspondence:** Belén Gago: [bgago@uma.es](mailto:bgago@uma.es)

# Supplementary Figures and Tables

Table 1. English version of the questionnaire for secondary education students.

| Exploring the human body | | | | |
| --- | --- | --- | --- | --- |
| We kindly ask you to answer the following questions to know what you have learned. It would help us to improve the workshop. Thank you very much! | | | | |
| Secondary school |  | Grade |  |  |
| Age |  | | | |
| Gender | | | | |
| Female | Male | Other |  | |
| Please cite the 3 main things you have learned | | | | |
|  | | | | |
| What would you like to have been explained that has not been mentioned? | | | | |
|  | | | | |
| Rate the station “biomolecules” (1 very poor, 5 excellent) | | | | |
| 1 | 2 | 3 | 4 | 5 |
| Rate the station “eukaryotic cells” (1 very poor, 5 excellent) | | | | |
| 1 | 2 | 3 | 4 | 5 |
| Rate the station “tissues” (1 very poor, 5 excellent) | | | | |
| 1 | 2 | 3 | 4 | 5 |
| Rate the station “organs” (1 very poor, 5 excellent) | | | | |
| 1 | 2 | 3 | 4 | 5 |
| Rate the station “systems” (1 very poor, 5 excellent) | | | | |
| 1 | 2 | 3 | 4 | 5 |
| Evaluate Mentor 1: | | | | |
| 1 | 2 | 3 | 4 | 5 |
| Evaluate Mentor 2: | | | | |
| 1 | 2 | 3 | 4 | 5 |
| Suggestions | | | | |

Table 2. Spanish version of the questionnaire for secondary education students.

| Explorando el cuerpo humano | | | | |
| --- | --- | --- | --- | --- |
| Para saber que has aprendido, y para que nosotros podamos mejorar la actividad, te pedimos que por favor rellenes las siguientes cuestiones. ¡Muchas gracias! | | | | |
| Centro educativo |  | Curso académico |  |  |
| Edad |  | | | |
| Sexo | | | | |
| Mujer | Hombre | Otro |  | |
| De todo lo que has aprendido, cita las 3 cosas que consideres más importantes. | | | | |
|  | | | | |
| ¿Qué te gustaría que se hubiera explicado y no ha sido mencionado? | | | | |
|  | | | | |
| Valora la estación “biomoléculas” (1 mínimo, 5 máximo) | | | | |
| 1 | 2 | 3 | 4 | 5 |
| Valora la estación “células eucariotas” (1 mínimo, 5 máximo) | | | | |
| 1 | 2 | 3 | 4 | 5 |
| Valora la estación “tejidos” (1 mínimo, 5 máximo) | | | | |
| 1 | 2 | 3 | 4 | 5 |
| Valora la estación “órganos” (1 mínimo, 5 máximo) | | | | |
| 1 | 2 | 3 | 4 | 5 |
| Valora la estación “sistemas” (1 mínimo, 5 máximo) | | | | |
| 1 | 2 | 3 | 4 | 5 |
| Valora al Mentor 1: | | | | |
| 1 | 2 | 3 | 4 | 5 |
| Valora al Mentor 2: | | | | |
| 1 | 2 | 3 | 4 | 5 |
| Sugerencias | | | | |

Table 3. English version of the questionnaire for undergraduate students.

| Exploring the human Body | | | | |
| --- | --- | --- | --- | --- |
| Please answer the following questions regarding the workshop, personal abilities and capacities, academic education and personal satisfaction | | | | |
| I consent to the use of my responses in this survey for the research study (ethical protocol 111-2023-H) | | | | |
| Yes | No |  | | |
| Gender | | | | |
| Female | Male | Other |  | |
| Had prior knowledge and background to participate in the workshop | | | | |
| Yes | No |  | | |
| I have received the necessary information to develop the different stations | | | | |
| Yes | No |  | | |
| Rate the station “biomolecules” (1 very poor, 5 excellent) | | | | |
| 1 | 2 | 3 | 4 | 5 |
| Rate the station “eukaryotic cells” (1 very poor, 5 excellent) | | | | |
| 1 | 2 | 3 | 4 | 5 |
| Rate the station “tissues” (1 very poor, 5 excellent) | | | | |
| 1 | 2 | 3 | 4 | 5 |
| Rate the station “organs” (1 very poor, 5 excellent) | | | | |
| 1 | 2 | 3 | 4 | 5 |
| Rate the station “systems” (1 very poor, 5 excellent) | | | | |
| 1 | 2 | 3 | 4 | 5 |
| The structure and length of time of the workshop seemed appropriate to me (1 strongly disagree, 5 strongly agree) | | | | |
| 1 | 2 | 3 | 4 | 5 |
| The material used in the stations was adequate (1 strongly disagree, 5 strongly agree) | | | | |
| 1 | 2 | 3 | 4 | 5 |
| Participation in this workshop has a positive impact on my academic training (1 totalmente en desacuerdo, 5 totalmente de acuerdo) | | | | |
| 1 | 2 | 3 | 4 | 5 |
| Participation in the workshop has helped me improve the following ability or skill | | | | |
| Team work | Oral communication skills | Self-steam | Information management | Autonomus work |
| Interpersonal relations | |  | | |
| Extracurricular activities are relevant to the academic training of an undergraduate student (1 strongly disagree, 5 strongly agree) | | | | |
| 1 | 2 | 3 | 4 | 5 |
| I would have liked to participated in this type of activity when I was a secondary school student | | | | |
| Sí | No |  | | |
| I have participated in similar activities before | | | | |
| Yes | No |  | | |
| More activities like this should be done at the University (1 strongly disagree, 5 strongly agree) | | | | |
| 1 | 2 | 3 | 4 | 5 |
| Overall, the workshop has far exceeded my expectations (1 strongly disagree, 5 strongly agree) | | | | |
| 1 | 2 | 3 | 4 | 5 |
| I would participate in the workshop again | | | | |
| Yes | No |  | | |
| I would recommend to my peers the participation in the workshop | | | | |
| Yes | No |  | | |
| Suggestions (things to improve, issues that could be included, etc) | | | | |

Table 4. Spanish version of the questionnaire for undergraduate students.

| Explorando el cuerpo humano | | | | |
| --- | --- | --- | --- | --- |
| Responde por favor a las siguientes preguntas sobre la actividad, habilidades y capacidades personales, formación académica y el grado de satisfacción personal | | | | |
| Doy mi consentimiento para el uso de mis respuestas en esta encuesta para el estudio de investigación (protocolo ético 111-2023-H) | | | | |
| Sí | No |  | | |
| Sexo | | | | |
| Mujer | Hombre | Otro |  | |
| Tenía conocimientos previos para participar en el taller | | | | |
| Sí | No |  | | |
| He recibido la información necesaria para desarrollar las diferentes estaciones | | | | |
| Sí | No |  | | |
| Valora la estación “biomoléculas” (1 mínimo, 5 máximo) | | | | |
| 1 | 2 | 3 | 4 | 5 |
| Valora la estación “células eucariotas” (1 mínimo, 5 máximo) | | | | |
| 1 | 2 | 3 | 4 | 5 |
| Valora la estación “tejidos” (1 mínimo, 5 máximo) | | | | |
| 1 | 2 | 3 | 4 | 5 |
| Valora la estación “órganos” (1 mínimo, 5 máximo) | | | | |
| 1 | 2 | 3 | 4 | 5 |
| Valora la estación “sistemas” (1 mínimo, 5 máximo) | | | | |
| 1 | 2 | 3 | 4 | 5 |
| La estructura y extensión en el tiempo de la actividad me han parecido adecuadas (1 totalmente en desacuerdo, 5 totalmente de acuerdo) | | | | |
| 1 | 2 | 3 | 4 | 5 |
| El material usado en las estaciones era adecuado (1 totalmente en desacuerdo, 5 totalmente de acuerdo) | | | | |
| 1 | 2 | 3 | 4 | 5 |
|  |  |  |  |  |
| La participación en esta actividad tiene una repercusión positiva en mi formación académica (1 totalmente en desacuerdo, 5 totalmente de acuerdo) | | | | |
| 1 | 2 | 3 | 4 | 5 |
| La participación en la actividad me ha ayudado a mejorar en las siguientes capacidades o habilidades | | | | |
| Trabajo en equipo | Comunicación oral | Autoestima | Gestión de la información | Trabajo autónomo |
| Relaciones interpersonales (empatía, tacto y escucha) | |  | | |
| Las actividades extraacadémicas tienen relevancia en la formación académica de un estudiante de grado (1 totalmente en desacuerdo, 5 totalmente de acuerdo) | | | | |
| 1 | 2 | 3 | 4 | 5 |
| Me hubiese gustado haber recibido este tipo de actividad como alumno de ESO o Bachillerato | | | | |
| Sí | No |  | | |
| He participado en actividades similares anteriormente | | | | |
| Sí | No |  | | |
| Se deberían llevar a cabo más actividades como esta desde nuestra Facultad (1 totalmente en desacuerdo, 5 totalmente de acuerdo) | | | | |
| 1 | 2 | 3 | 4 | 5 |
| En su conjunto, la actividad ha respondido a mis expectativas (1 totalmente en desacuerdo, 5 totalmente de acuerdo) | | | | |
| 1 | 2 | 3 | 4 | 5 |
| Participaría de nuevo en la actividad | | | | |
| Sí | No |  | | |
| Recomendaría la participación en ella a compañeros | | | | |
| Sí | No |  | | |
| Sugerencias (cosas que mejorar y cómo mejorarlas, temas que se podrían tratar, etc) | | | | |
